# Pharmacodynamic Modeling to Evaluate the Impact of Cimetidine, an OCT2 Inhibitor, on the Anticancer Effects of Cisplatin

**DOI:** 10.3390/cells12010057

**Published:** 2022-12-23

**Authors:** Hardik Mody, Tanaya R. Vaidya, Lawrence J. Lesko, Sihem Ait-Oudhia

**Affiliations:** 1 Center for Pharmacometrics and Systems Pharmacology, Department of Pharmaceutics, College of Pharmacy, University of Florida, Orlando, FL 32827, USA; 2 Quantitative Pharmacology and Pharmacometrics (QP2), Merck & Co., Inc, Kenilworth, NJ 07033, USA

**Keywords:** cisplatin, cimetidine, modeling, OCT2, drug–drug interaction

## Abstract

Despite potent anticancer activity, the clinical utilization of cisplatin is limited due to nephrotoxicity. As Organic Cation Transporter 2 (OCT2) has been shown to be one of the key transporters involved in the uptake of cisplatin into renal proximal tubules, OCT2 inhibitors such as cimetidine have been explored to suppress cisplatin-induced nephrotoxicity. Nonetheless, the impact of OCT2 inhibition or cimetidine on the anti-cancer effects of cisplatin has not been extensively examined. The main objective of the present study was to quantitatively characterize the anticancer effects of cisplatin and cimetidine and determine their nature of interactions in two cancer cell lines, OCT2-negative hepatocellular carcinoma (HCC) cell line, Huh7, and OCT2-positive breast cancer cell line, MDA-MB-468. First, we determined the static concentration-response curves of cisplatin and cimetidine as single agents. Next, with the help of three-dimensional (3D) response surface analyses and a competitive interaction model, we determined their nature of interactions at static concentrations to be modestly synergistic or additive in Huh7 and antagonistic in MDA-MB-468. These results were consistent with the cell-level pharmacodynamic (PD) modeling analysis which leveraged the time-course effects of drugs as single agents and drug combinations. Our developed PD model can be further used to design future preclinical studies to further investigate the cisplatin and cimetidine combinations in different in vitro and in vivo cancer models.

## 1. Introduction

Cisplatin is one of the most potent chemotherapeutic agents and is widely used for the treatment of a variety of cancer types. However, its clinical use is limited due to severe side effects, including nephrotoxicity, ototoxicity, and peripheral neurotoxicity [1,2,3]. Of them, nephrotoxicity continues to be a major concern as it occurs in greater than 30–40% of the patients undergoing cisplatin treatment and is often dose-limiting [1,3]. Accumulating studies have indicated the role of the organic cation transporter 2 (OCT2), a transporter which is present on the basolateral membrane of the kidney proximal tubule cells, in the renal secretion or kidney uptake of cisplatin [4,5,6,7]. Thus, cisplatin-induced nephrotoxicity is attributed to active drug transport and accumulation into the renal epithelial cells, which subsequently leads to nuclear and mitochondrial DNA damage, cell apoptosis and necrosis, progressively evolving from an acute kidney injury (AKI) to a chronic disease and ultimately to kidney failure. Hence, targeting transporters such as OCT2 is sought to be a viable approach to inhibit kidney uptake and thereby overcome cisplatin-induced nephrotoxicity, at least partially if not fully [4,8,9,10]. 

Cimetidine is an OCT2 substrate and competitively inhibits the cellular uptake of cisplatin via OCT2 transporters. Therefore, the combination of cimetidine with cisplatin therapy has been investigated to potentially reduce cisplatin-induced nephrotoxicity [4,8,10]. Cimetidine co-treatment consistently reduced the in vitro toxicity mediated by cisplatin in freshly isolated human proximal tubule cells expressing OCT2 [4]. Similarly, in vivo studies showed that the administration of cisplatin resulted in acute renal tubular necrosis in the kidneys of wildtype mice but not in OCT2 knockout mice [8]. In addition, co-treatment with cimetidine decreased the nephrotoxic effects of cisplatin in the wildtype mouse model [8]. 

While targeting OCT2 seems like an attractive strategy to circumvent cisplatin-induced nephrotoxicity, it is imperative to ensure that such a strategy does not compromise the anticancer efficacy of cisplatin. For instance, OCT2 inhibition or co-treatment with cimetidine can block, at least partially if not fully, the transport of cisplatin into cancer cells (especially those expressing OCT2), thereby suppressing its activity. While the nephroprotective effects of cimetidine have been extensively characterized in preclinical studies, very few studies have investigated the impact of cimetidine on the anticancer effects of cisplatin [10,11]. Besides, previous studies have demonstrated anticancer effects of cimetidine alone via apoptosis in multiple cancer models [12,13,14,15]. Therefore, it is important to evaluate the combinatorial effects of cisplatin and cimetidine (hereafter referred to as CIS and CIM in the manuscript) on the overall anticancer efficacy. 

The main objective of the current study is to quantitatively characterize the anticancer effects of CIS and CIM as single agents and combinations in two cancer cell lines, OCT2-negative liver cancer cell line, Huh7, and OCT2-positive breast cancer cell line, MDA-MB-468, using a combination of experimental data and in vitro pharmacodynamic (PD) modeling approaches. First, the effects of CIS and CIM as single agents in both the cancer cell lines were evaluated with the help of concentration-response curves and by determining their respective in vitro half-maximal inhibitory concentrations (IC_50_s). Based on this, CIS and CIM concentrations were selected for static and time-course drug combination studies. Next, the static combinatorial effects of CIS and CIM and the nature of their interactions in the two cancer cell lines were determined with the help of a three-dimensional (3D) response surface analysis using a competitive interaction model. Finally, the time-course effects of the CIS, CIM and their combinations were determined with the help of a mathematical in vitro pharmacodynamic (PD) model, and relevant parameters including interaction parameters were estimated. Thus, with the help of experimental and PD modeling approaches, the impact of OCT2 inhibition or co-treatment with cimetidine on the anticancer effects of cisplatin was evaluated in human cancer cell lines.

## 2. Materials and Methods

### 2.1. Drugs, Reagents, and Cell Line 

The human hepatocellular carcinoma (HCC) cell line, Huh7, and breast cancer cell line, MDA-MB-468 cells were purchase from American Type Culture Collection (ATCC) (Manassas, VA, USA). Both cell lines were cultured, maintained, passaged, and used per manufacturer’s instructions and as extensively described previously [16,17]. 

Cisplatin and cimetidine were purchased from Millipore Sigma-Aldrich Co. (St. Louis, MO). Cimetidine was dissolved in molecular biology grade water while sodium chloride (NaCl) solution in water was used for cisplatin as per manufacturer’s instructions. While cimetidine stock solutions were stored at −80 °C, but cisplatin stocks were always prepared fresh and stored, if required in dark at 2–8 °C. Fresh serial dilutions from the drug stocks were prepared each time prior to experiments.

Cell culture reagents including Dulbecco’s Modified Eagle’s Medium (DMEM), MEM non-essential amino acids, sodium bicarbonate, Penicillin/Streptomycin, Phosphate Buffered Saline (PBS), molecular biology grade water and 0.25% trypsin/2.21 mM EDTA were purchased from Corning (Corning, NY, USA). Fetal Bovine Serum (FBS) and Cell Counting kit-8 (CCK-8) were purchased from Millipore Sigma-Aldrich (St. Louis, MO, USA). 

### 2.2. CCK-8 Cell Viability Assay

Based on growth patterns, Huh7 and MDA-MB-468 cells were seeded at a density of 5 × 10^3^ cells per well (100 µL) of a 96-well plate. After overnight incubation, the cell lines were exposed to varying concentrations of CIS (0.001 to 25 µM), CIM (0.05 to 10 mM), or their combinations for 72 to 96 h for various sets of experiments. The CCK8 cell viability assay was subsequently carried out at different time points as indicated per the manufacturer’s instructions. Briefly, cells are incubated in CCK-8 solution (10 µL/well of a 96-well plate) for an hour with absorbance measured at 450 nm using a microplate spectrophotometer Biotek (Winooski, VT, USA). Experiments were performed in at least triplicates and compared against controls. 

### 2.3. Mathematical Modeling

#### 2.3.1. Concentration-Response Relationships and Determination of IC_50_

An inhibitory Hill function was used to characterize the concentration-response curves. The maximal inhibitory effects (*I_max_*) and concentrations required to achieve 50% of maximal effects (*IC_50_*) were estimated for CIS and CIM as single agents in the two human cancer cell lines, Huh7 and MDA-MB-468, at 72 h:

(1) $$R=R_{0}\;.\;\left(1-\frac{I_{max}\;.\;C^{\gamma }}{IC_{50}^{\gamma }+C^{\gamma }}\right)$$

where *R* is the response to drug treatments (% cell viability), *R_0_* is the baseline response (% cell viability in absence of drug treatments or control), *I_max_* is the maximal inhibitory effect of drug treatments, *IC_50_* is the drug concentration required to achieve 50% of *I_max_*, *C* is the drug concentration and γ is the Hill coefficient (Table 1). The concentration-response curves were used to select concentrations for single agents and combinations during time-course and subsequent studies. 

#### 2.3.2. Evaluation of Static Concentration-Response Drug-Drug Interactions 

A competitive interaction model [18,19] was used to evaluate the static combinatorial effects of CIS and CIM on the cell viability of Huh7 and MDA-MB-468 cells. The model was fitted to the concentration-response data for the treatment combination as follows:

(2) $$R=R_{0}\;.\;\left[1-\frac{\left(I_{max,A}.{\left(\frac{C_{A}}{\psi .IC50_{A}}\right)}^{\gamma_{A}}\right)+\left(I_{max,B}.{\left(\frac{C_{B}}{\psi .IC50_{B}}\right)}^{\gamma_{B}}\right)}{{\left(\frac{C_{A}}{\psi .IC50_{A}}\right)}^{\gamma_{A}}+{\left(\frac{C_{B}}{\psi .IC50_{B}}\right)}^{\gamma_{B}}+1}\right]$$

where, *R* is % cell viability, *R*_0_ is % cell viability at baseline (i.e., 100%), *I_max,A_* and *I_max,B_* are the maximal effects of the two drugs (i.e., maximal fractions of inhibition), *C_A_* and *C_B_* are concentrations of the drugs, *IC*_50*A*_ and *IC*_50*B*_ are the half-maximal inhibitory concentrations of the drugs, *γ_A_* and *γ_B_* are the respective Hill coefficients and *ψ* is the interaction term. All individual drug-related parameters were fixed from the CIS and CIM concentration-response curve fittings and the interaction term, ψ, was estimated. The apparent interaction between the drugs was antagonistic when *ψ* > 1, synergistic when *ψ* < 1 and additive when *ψ* = 1. Finally, three-dimensional (3D) response surface plots of cell viability versus CIS and CIM concentrations were constructed to visually evaluate the interaction between the drugs for both cell lines. An additive interaction was assumed between both drugs (i.e., *ψ* = 1) and 3D response surface plots were generated for both cell lines using Equation (2) with MATLAB version 2017a The MathWorks, Inc. (Natick, MA, USA). Finally, observed cell viability data were overlaid on the additive response surface plots to assess location of the data points relative to the additive surface, thus enabling visual assessment of additive, antagonistic or synergistic effects between CIS and CIM in Huh7 and MDA-MB-468. 

### 2.4. Development of the In Vitro Cellular Level Pharmacodynamic (PD) Response Model 

The in vitro cellular level pharmacodynamic model (PD) was developed to evaluate the effects for the single agents, CIS, CIM and their combinations on the cell viability of Huh7 and MDA-MB-468. The corresponding parameters, their definitions and estimated values are listed in Table 2. It was assumed that there was no change in drug concentrations and no degradation of drugs over time in cell culture media. In addition, it was assumed that the two drugs do not interact with each other in the cell culture media for their combination group. 

The cellular growth of Huh7 and MDA-MB-468 in the control group (no drug treatment) was best described by an exponential growth function as follows:

(3) $$\frac{dR}{dt}=k_{g}\;.\;R\;;\;R\;(0)=R_{0}$$

where *R* is the % cellular viability (cellular response) at time *t*, *k_g_* is the first-order growth rate constant for cancer cell lines, while *R*_0_ is the % cellular viability at time zero.

#### 2.4.1. Single-Agent PD Models 

The PD effect of CIS or CIM on the cell viability of Huh7 and MDA-MB-468 was described by a stimulatory effect (Hill function) on the cell death of cancer cells [20,21]. Three transit compartments were included in the model structure to capture the delayed in vitro drug cytotoxic effects in cancer cell lines. The delayed effects of CIS and CIM have been attributed to their mechanisms including a temporal delay due to the intracellular signaling cascade involved in their cytotoxic effects. 

The differential equations for the effect of CIS or CIM as single agents in Huh7 and MDA-MB-468 are as follows:

(4a) $$K_{x}=\frac{S_{max,x}\;.\;C_{x}}{SC_{50,x}+C_{x}}$$

(4b) $$\frac{dK1_{x}}{dt}=\frac{1}{\tau_{x}}\;.\left(K_{x}-K1_{x}\right)\;;\;K1_{x}\;(0)=0$$

(4c) $$\frac{dK2_{x}}{dt}=\frac{1}{\tau_{x}}\;.\left(K1_{x}-K2_{x}\right)\;;\;K2_{x}\;(0)=0$$

(4d) $$\frac{dK3_{x}}{dt}=\frac{1}{\tau_{x}}\;.\left(K2_{x}-K3_{x}\right)\;;\;K3_{x}\;(0)=0$$

(4e) $$\frac{dR}{dt}=k_{g}\;.\;R-K3_{x}\;.\;R\;\;;\;R(0)=R_{0}$$

where the subscript *x* represents either CIS or CIM, *S_max,x_* is the maximal killing rate constant while *SC*_50,*x*_ is the concentration of CIS or CIM required to induce half-maximal cell-killing effect in Huh7 and MDA-MB-468. *K_x_* is the cytotoxicity Hill function, *K*1*_x_* to *K*3*_x_* are transit compartments with *Ʈ_x_* being the mean transit time between compartments and C*_x_* represents the drug concentration. 

#### 2.4.2. Drug Combination PD Models

Both drugs are assumed to exert cytotoxic effects (stimulation of death function) in Huh7 and MDA-MB-468 cancer cell lines. Hence, the PD model for the drug combinations include an interaction parameter, ψ, which was applied to Equation (5a), where ψ = 1, <1, or >1 indicate additive, synergistic or antagonistic interactions, respectively. The following differential equations were used to characterize the combinatorial effects of CIS and CIM in Huh7 and MDA-MB-468:

(5a) $$K_{CIS}=\frac{S_{max,CIS}\;.\;C_{CIS}}{(SC_{50,CIS}\;.\;\psi )+C_{CIS}}$$

(5b) $$\frac{dK1_{CIS}}{dt}=\frac{1}{\tau_{CIS}}\;.\left(K_{CIS}-K1_{CIS}\right)\;;\;K1_{\mathrm{CIS}}\;(0)=0$$

(5c) $$\frac{dK2_{CIS}}{dt}=\frac{1}{\tau_{CIS}}\;.\left(K1_{CIS}-K2_{CIS}\right)\;;\;K2_{\mathrm{CIS}}\;(0)=0$$

(5d) $$\frac{dK3_{CIS}}{dt}=\frac{1}{\tau_{CIS}}\;.\left(K2_{CIS}-K3_{CIS}\right)\;;\;K3_{\mathrm{CIS}}\;(0)=0$$

(5e) $$K_{CIM}=\frac{S_{max,CIM}\;.\;C_{CIM}}{SC_{50,CIM}+C_{CIM}}$$

(5f) $$\frac{dK1_{CIM}}{dt}=\frac{1}{\tau_{CIM}}\;.\left(K_{CIM}-K1_{CIM}\right)\;;\;K1_{\mathrm{CIM}}\;(0)=0$$

(5g) $$\frac{dK2_{CIM}}{dt}=\frac{1}{\tau_{CIM}}\;.\left(K1_{CIM}-K2_{CIM}\right)\;;\;K2_{\mathrm{CIM}}\;(0)=0$$

(5h) $$\frac{dK3_{CIM}}{dt}=\frac{1}{\tau_{CIM}}\;.\left(K2_{CIM}-K3_{CIM}\right)\;;\;K3_{\mathrm{CIM}}\;(0)=0$$

(5i) $$\frac{dR}{dt}=k_{g}\;.\;R-(K3_{CIS}+K3_{CIM})\;.\;R\;\;;\;R\;(0)=R_{0}$$

The time-course data of the single agents were characterized first with the single-agent PD models, parameters related to the single agents were estimated, and then fixed while estimating the interaction parameter and characterizing the drug combination data in the two cell lines. All model fittings and simulations were performed using Monolix suites version 2016R1 or higher (Antony, France: Lixoft SAS, 2016). 

## 3. Results

### 3.1. Determination of IC_50_ for CIS and CIM as Single Agents from Static Concentration-Response Curves

**Figure 1:**
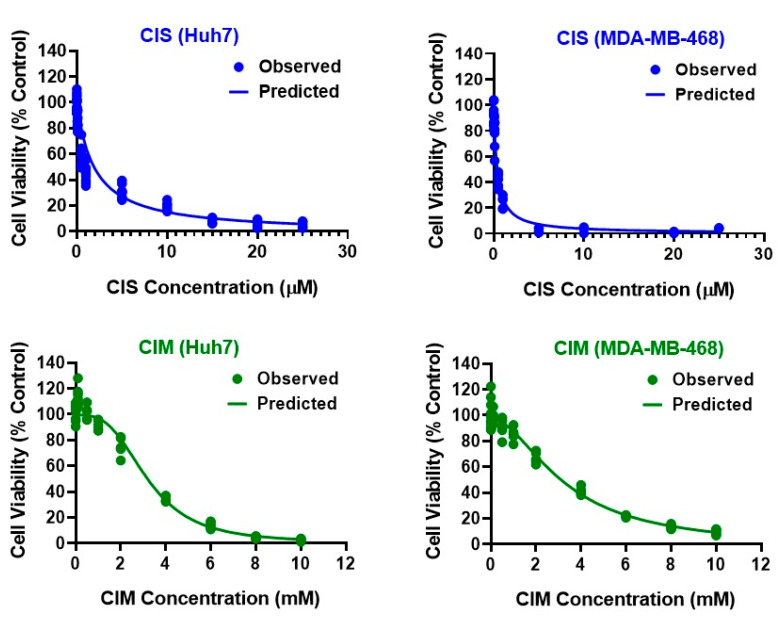
Concentration-response curves for CIS (**top**, blue) and CIM (**bottom**; green) as single agents in Huh7 (**left**) and MDA-MB-468 (**right**) cancer cell lines. Observed data are represented by solid circles while the smooth lines are model fittings.

The two cancer cell lines, Huh7 (OCT2-negative) and MDA-MB-468 (OCT2-positive), were treated with a wide range of either CIS (0.001 to 25 µM) or CIM (0.1 to 10 mM) concentrations for 72 h. The inhibitory Hill model was fitted to the static concentration-response curves of CIS and CIM as single agents in both the cell lines as shown in Figure 1 and parameter estimates are summarized in Table 1. As anticipated, CIS was consistently determined to be more potent as compared to CIM by ~1000-fold in Huh7 and ~10,000-fold in MDA-MB-468. The IC_50_s for CIS were 1.96 µM and 0.39 µM while that for CIM were 3.19 mM and 3.28 mM in Huh7 and MDA-MB-468, respectively. Thus, MDA-MB-468 was more sensitive towards CIS as compared to Huh7 (~ 5-fold), while both cell lines showed similar sensitivities for CIM as evidenced by their estimated IC_50_s in the respective cell lines. On the other hand, both CIS and CIM showed similar maximal killing (close to 1) for both the cancer cell lines. A hill coefficient was used to accurately characterize the concentration-response curves for CIM and was estimated at 3.12 and 1.83 for Huh7 and MDA-MB-468, respectively. The inhibitory Hill model captured the concentration-response data well, as demonstrated with observations versus individual prediction plots shown in Supplementary Figure S1. 

### 3.2. Static Concentration-Response Combinatorial Drug Effects of CIS and CIM 

**Figure 2:**
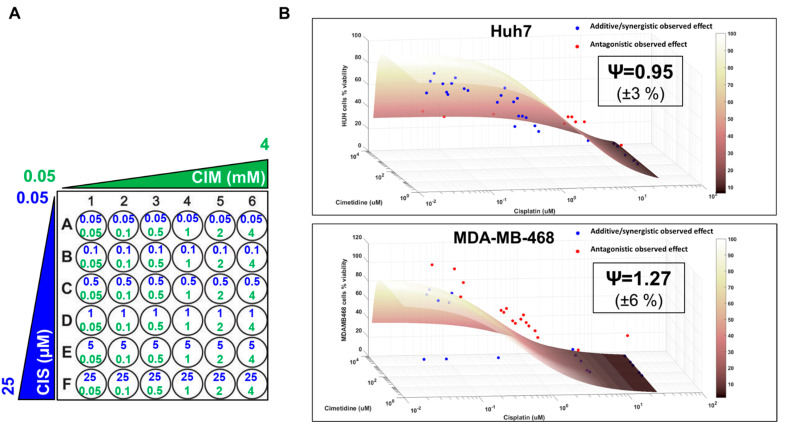
Three-dimensional response surface plots from the effects of single agents (CIS or CIM) and their combination data in Huh7 and MDA-MB-468 using a competitive interaction model. The concentrations used for single agents, CIS (0.05 to 25 µM) or CIM (0.05 to 4 mM) and their combinations are summarized in **A**. The 3D response surface plots represent model simulations under the assumption of an additive interaction (ψ = 1) while the circles represent observed data for Huh7 (**B**, top) and MDA-MB-468 (**B**, bottom), respectively. Red circles are above the surface and indicate antagonistic interactions while blue circles are at or below the surface and indicate additive or synergistic interactions between CIS and CIM. ψ = 0.95 and ψ = 1.27 indicate interaction parameters estimated using a competitive interaction model for Huh7 and MDA-MB-468 cells, respectively.

After evaluating the concentration-response curves of CIS and CIM as single agents, Huh7 and MDA-MB-468 were exposed to a range of CIS and CIM concentrations, both as single agents as well as their combinations for 72 h as indicated in Figure 2A. Six concentrations of CIS (from 0.05 µM to 25 µM), 6 concentrations of CIM (from 0.05 to 4 mM) and their 36 different combinations were used in both cancer cell lines. A competitive interaction model was fitted to the above data to examine the cancer cell killing effects of the drug combinations and to determine the nature of their interactions in the static setting. Based on this analysis, the interaction parameter, ψ, was estimated at 0.95 and 1.27 for Huh7 and MDA-MB-468 cells, respectively, which suggested that CIS and CIM combinations were modestly synergistic or additive (ψ slightly less than 1) for OCT2-negative cell line, Huh7 while antagonistic (ψ greater than 1) for OCT2-positive cell line, MDA-MB-468. In addition, 3D response surfaces were plotted to visually interpret and appreciate the differences of the CIS and CIM interactions in the two cell lines. The 3D plots were generated under the assumption of additive interaction (ψ = 1) and observed data were overlaid on the plots (Figure 2B). The nature of CIS and CIM interaction was also reflected in the 3D plots (Figure 2B), wherein, most of the observed data points lay below or at the additive response surface for Huh7, indicating a mildly synergistic or additive effect. On the other hand, several observed data points were above the additive surface indicating antagonistic interactions for CIS and CIM in MDA-MB-468 cells. These observations were consistent with the interaction parameter estimates from the competitive interaction model in the two cell lines. 

### 3.3. Time Course Effects of CIS and CIM Single Agents and Combination in Human Cancer Cell Lines

**Figure 3:**
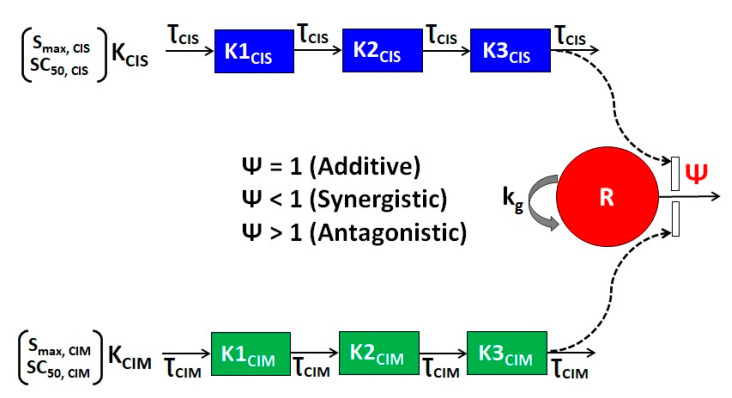
Schematic of the in vitro cellular-level pharmacodynamic model (PD) for the single agents CIS and CIM and their combination effects on human cancer cell lines, Huh7 and MDA-MB-468. All the model parameters and their definitions are listed in Table 2. Solid lines with arrows denote turnover of the indicated response. The red circle represents cell viability while the blue or green boxes represent transit compartments to describe the delay in the effects of CIS or CIM, respectively. The open solid rectangles represent the stimulation of death induced by CIS, CIM as single agents or by their combination as indicated by dashed black arrows. The interactions of CIS and CIM on the stimulation of death of cancer cells is captured by ψ with ψ = 1, ψ < 1, or ψ > 1 indicating additive, synergistic, or antagonistic interactions, respectively.

**Figure 4:**
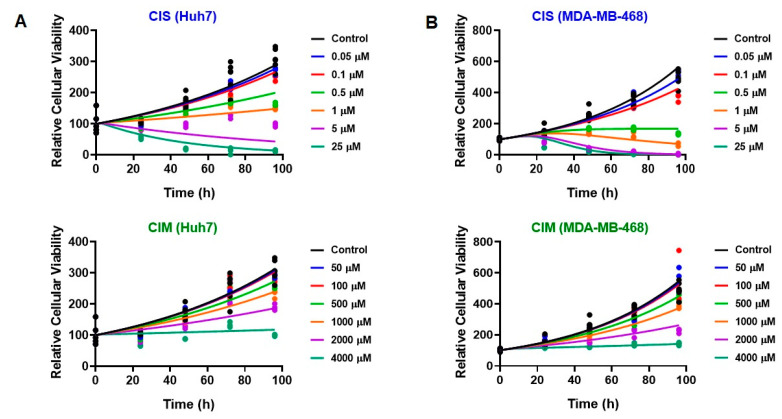
(**A,B**) Model fittings (using in vitro cell-level PD model) for the in vitro effects of the single agents, CIS (top) or CIM (bottom) at the indicated concentrations over time on the cell viability of human cancer cell lines, Huh7 (**A**) and MDA-MB-468 (**B**). Solid circles represent observed data while smooth lines are model fittings.

The time course effects of CIS and CIM as single agents as well as their combinations on the cell viability of Huh7 and MDA-MB-468 was characterized with the help of an in vitro cellular level PD response model as shown in Figure 3. As with the static concentration studies, six concentrations of CIS (from 0.05 µM to 25 µM) or six concentrations of CIM (from 0.05 to 4 mM) were used as single agents with the time-course data up to 96 h. In addition, four concentrations of CIS (from 0.05 µM to 1 µM), each were combined with the six concentrations of CIM (from 0.05 to 4 mM) to form 24 different CIS and CIM combinations which were evaluated in both the cell lines. As shown in Figure 4, saturation of responses was observed at the highest concentrations of CIS as a single agent in both the cell lines, and hence not used for drug combination studies. 

**Figure 5:**
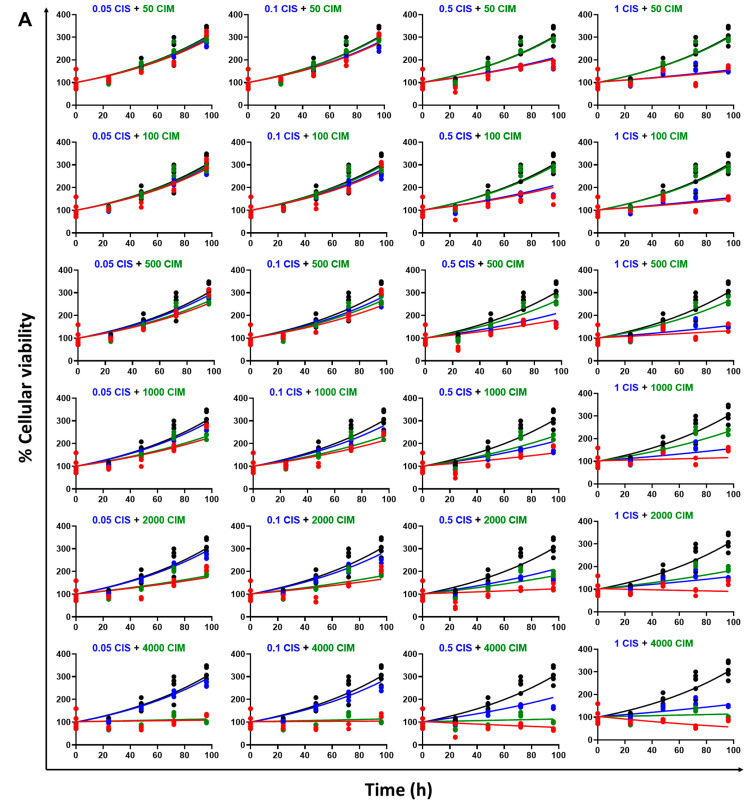

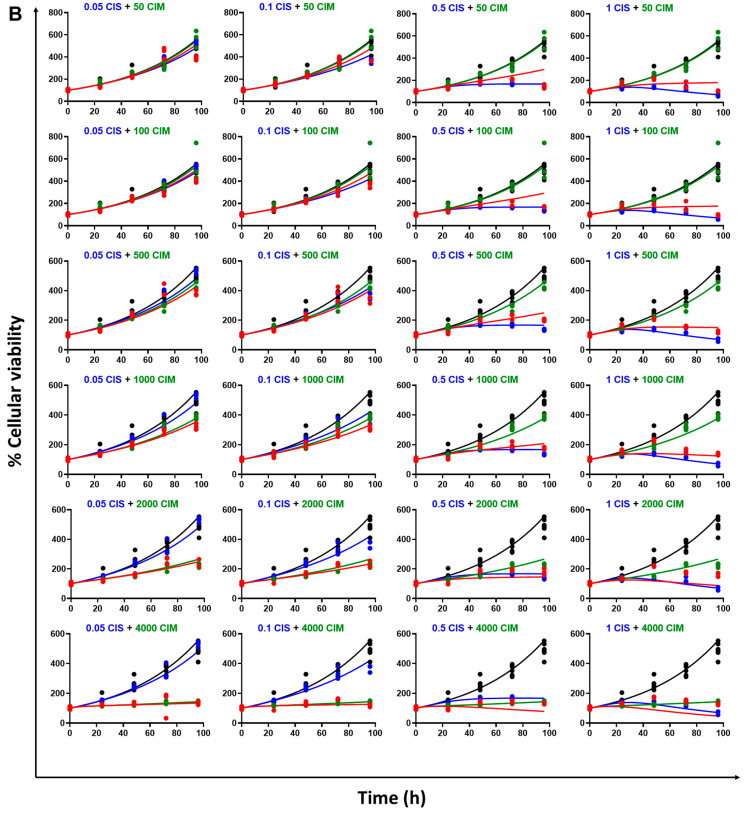
Model fittings (using in vitro cell-level PD model) for the in vitro effects of CIS and CIM as single agents or their combination at the indicated concentrations over time on the cell viability of Huh7 (**A**) and MDA-MB-468 (**B**). Solid circles represent observed data while smooth lines are model fittings. Black, Control; Blue, CIS; Green, CIM; Red, CIS+CIM. The concentrations (in µM) used are indicated at the top of each graph for single agents and combinations.

The model fittings for the time-course effects of CIS or CIM as single agents in Huh7 and MDA-MB-468 are shown in Figure 4A and Figure 4B, respectively. In addition, the model fittings for the drug combinations and comparison with the single agents’ profiles are shown in Figure 5A for Huh7 and in Figure 5B for MDA-MB-468. Overall, the model was able to simultaneously capture most of the data relatively well, as demonstrated by the model fits, except for the 5 µM CIS treatment arm for Huh7, where the model predictions overestimated the cell killing. However, the overall observations versus individual predictions plot shows roughly uniform distribution of the observed data around the line of identity, thereby suggesting that the model generally characterized the data well (Supplementary Figure S2). In addition, the model-based parameters, as summarized in Table 2, were estimated with good precision.

The cell viability of cancer cell lines (in absence of drug treatments) was first characterized with an exponential growth function (Equation (3)) using a first-order growth rate constant, kg which was estimated to be 0.011 (±3.32%) h^−1^ and 0.018 (±1.4%) h^−1^, for Huh7 and MDA-MB-468, respectively. The time course effects of CIS or CIM on the cell viability of cancer cell lines over 96 h was adequately captured with the stimulatory effects on cell death characterized with a capacity-limited, Hill function (Equations (4a)–(4e)). The estimated, maximal killing rate constant (*S_max,CIS_*) for CIS was 0.038 (± 9.63%) h^−1^ and 0.108 (± 5.75%) h^−1^ while the CIS concentration inducing 50% of maximal cell killing rate (*SC_*50,*CIS_*) was estimated to be 4.27 (± 6.32%) µM and 2.48 (± 8.13%) µM for Huh7 and MDA-MB-468, respectively. On the other hand, the estimated maximal killing rate constant (S_max, CIM_) for CIM was 0.106 (± 2.25%) h^−1^ and 0.096 (± 17.1%) h^−1^ while the CIM concentration inducing 50% of maximal cell killing rate (*SC_*50,*CIM_*) was estimated to be ~37 (± 22.7%) mM and ~21 (± 21.3%) mM for Huh7 and MDA-MB-468, respectively. Thus, time-course data analysis also confirmed CIS to be far more potent as compared to CIM (~10,000-fold based on respective *SC*_50_ estimates) which is consistent with the concentration-response analysis. The delayed effects of CIS and CIM were captured well with the help of three transit compartments (*K*1 to *K*3) on the stimulation of death function, with *Ʈ_CIS_* and *Ʈ_CIM_* representing the mean transit time. 

Next, the time-course combinatorial effects of CIS and CIM on the cell viability or stimulation of death of Huh7 and MDA-MB-468 cell lines was characterized by fixing the parameter estimates of single agents and incorporating an interaction parameter ψ in the model structure as shown in Figure 3. The nature of interaction is interpreted as additive, synergistic, or antagonistic if ψ = 1, ψ < 1, or ψ > 1, respectively (Equations (5a)–(5i)). The model-based analysis estimated ψ as 0.96 and 2.2 indicating slightly synergistic/additive and antagonistic interactions for CIS and CIM for OCT2-negative cell line, Huh7, and OCT2-positive cell line, MDA-MB-468 cell lines, respectively. This is largely consistent with the competitive-interaction model-based 3D response surface analysis discussed previously (Figure 2). 

## 4. Discussion

Despite its high antitumor efficacy, the clinical utilization of CIS is limited due to organ toxicities including nephrotoxicity, which can often be dose-limiting [1,2,3]. Previous studies have shown that OCT2 is one of the key transporters involved in the uptake of CIS into the renal proximal tubules which ultimately can lead to drug accumulation and renal toxicities [4,5,6,7]. Hence, combination of CIS with an OCT2 inhibitor, CIM has been previously explored to overcome CIS-induced nephrotoxicity with several in vitro and in vivo studies showing promising results [4,8,10]. However, CIM can also potentially impact the anticancer activity of CIS, especially in OCT2 expressing cancer cells and hence further investigation is warranted. In the present study, we sought to determine the impact of CIM on the anticancer effects of CIS and quantitatively characterize their nature of interactions in two cancer cells, OCT2-negative HCC cell line, Huh7, and OCT2-positive breast cancer cell line MDA-MB-468. First, we quantitatively captured the static concentration response curves as well as time-course anti-cancer activity profiles for CIS and CIM as single agents. Subsequently, we characterized their nature of interactions in the cancer cell lines with the help of mathematical modeling and estimating an interaction parameter, ψ. The results suggest that the combinatorial effects of CIS and CIM were additive or slightly synergistic in OCT2-negative Huh7 while antagonistic in OCT2 expressing MDA-MB-468. 

The breast cancer cell line, MDA-MB-468 was previously reported to express high levels of OCT2 protein [22,23], hence was deemed an OCT2-positive cell line for the present study. On the other hand, human liver, and liver-originating HCC cell lines, including Huh7, was previously implicated for absence of OCT2 [24,25,26] and hence was deemed an OCT2-negative cell line for the present study. In addition, both cell lines were also selected due to previous reports demonstrating their sensitivity towards CIS treatment [27,28]. Interestingly, MDA-MB-468 (IC_50_ = 0.39 µM) demonstrated higher sensitivity towards CIS as compared to Huh7 (IC_50_ = 1.96 µM) by ~5-fold which could be explained, at least partially, due to higher OCT2 expression levels in the former as compared to the latter. 

In addition, we observed that CIM alone also mediated cytotoxicity in the cancer cell lines, although at much higher concentrations (Figure 1). This is consistent with previous studies showing anticancer effects of CIM via apoptosis in multiple in vitro and/or in vivo cancer models including gastric, pancreatic, and cholangiocarcinoma [12,13,14,15]. The in vitro IC_50_s for CIM as a single agent were estimated at 3.28 mM and 3.19 mM in MDA-MB-468 and Huh 7, respectively (which is ~10,000 and 1000-folds higher as compared to that of CIS) which are consistent with previous reports [12,13,14,15]. Besides, high CIM concentrations are also required while mediating nephroprotective activity against CIS as demonstrated by previous studies [11]. Based on the above considerations and IC_50_ estimates, a lower range of CIS concentrations (0.05 µM to 25 µM) and a higher range of CIM concentrations (from 0.05 to 4 mM) were used for subsequent static combination studies. With the help of a competitive interaction model and 3D response-surface analyses of the static single agent and combination profiles, the nature of interaction was determined as slightly synergistic or additive for CIS and CIM in Huh7 but antagonistic in MDA-MB-468 (Figure 2). Finally, we developed a cell-level PD model to evaluate the time-course effects of CIS and CIM as single agents as well as their combinatorial effects in cancer cells. As with the above model-based analysis from the static experiments, the cell-level PD model also determined CIS and CIM interactions to be slightly synergistic or additive in Huh7 (ψ = 0.96) while antagonistic in MDA-MB-468 (ψ = 2.2). 

Finally, few studies have previously reported that CIM did not impact the antitumor effects of CIS in vitro or in vivo in OCT2 expressing cancer models; however, those studies evaluated the impact of CIM at only one or two concentrations or dose levels [10,11]. Hence, this is the first study to comprehensively evaluate the impact of OCT2 inhibition or cimetidine on the in vitro anticancer effects of CIS across multiple concentrations and time points, and to systemically examine their nature of interactions at static concentrations and over a time-course using multiple modeling approaches in OCT2 expressing and non-expressing cancer cell lines.

## 5. Conclusions

Taken together, the above results indicate that combining CIM can potentially be beneficial in improving anticancer activity of CIS, especially in OCT2-negative cancer cells. Future studies should also aim to investigate the underlying mechanisms contributing to the synergistic interactions of CIS and CIM in OCT2-negative cancer cell lines. While antagonistic interactions were determined in the OCT2-positive cancer cells, it should be noted that the combination can still be beneficial to patients with OCT2-positive tumors in suppressing CIS-induced nephrotoxicity, especially at higher doses or for a longer duration and thereby benefit from the anticancer activity of CIS. Our developed cell-level PD model can be further used to better design future preclinical studies to further investigate the impact of OCT2 inhibition on the effects of cisplatin in different in vitro and in vivo cancer models. 

## Figures and Tables

**Table 1:** Concentration-response curve parameter estimates for CIS and CIM as single agents in Huh7 and MDA-MB-468 cancer cell lines. % RSE = % relative standard error in the model parameters.

Parameter (Units)	Definition	Estimate (% RSE)			
		CIS Huh7	CIS MDA-MB-468	CIM Huh7	CIM MDA-MB-468
R_0_ (%)	Baseline % Cell Viability	100 (Fixed)	100 (Fixed)	99.5 (2.87)	97.4 (2.17)
IC_50_ (µM or mM)	Drug concentration inducing 50% of maximal effect	1.96 µM (9.82)	0.39 µM (11.8)	3.19 mM (4.66)	3.28 mM (3.85)
I_max_	Maximal effect	1 (0.99)	1 (Fixed)	0.99 (0.74)	1 (2.78)
γ	Hill coefficient	1 (Fixed)	1 (Fixed)	3.12 (9.87)	1.83 (10.5)

**Table 2:** Parameter estimates for the in vitro cellular-level pharmacodynamic model (PD) for the single and combinatorial effects of CIS and CIM on Huh7 and MDA-MB-468. % RSE = % relative standard error in the model parameters.

Parameter (Units)	Definition	Estimate (% RSE)	
		Huh7	MDA-MB-468
*R*_0_ (%)	Baseline % cell viability	100 (Fixed)	100 (Fixed)
*k_g_* (h^−1^)	First-order growth rate constant	0.011 (3.32)	0.018 (1.4)
*S_max,CIS_* (h^−1^)	Maximal killing rate constant of CIS	0.038 (9.63)	0.108 (5.75)
*SC_50,CIS_* (µM)	CIS concentration inducing 50% of maximal killing rate	4.27 (6.32)	2.48 (8.13)
1/*Ʈ_CIS_* (h^−1^)	Transit constant for the stimulation of death by CIS	1.17 (0.565)	0.101 (7.94)
*S_max,CIM_* (h^−1^)	Maximal killing rate constant of CIM	0.106 (2.25)	0.096 (17.1)
*SC_50,CIM_* (µM)	CIM concentration inducing 50% of maximal killing rate	36,886.97 (22.7)	21,453.95 (21.3)
1/*Ʈ_CIM_* (h^−1^)	Transit constant for the stimulation of death by CIM	1.92 (0.698)	0.523 (4.69)
*ψ*	Interaction parameter	0.96 (0.017)	2.2 (4.24)

## Data Availability

Not applicable.

## Supplementary Materials

The following supporting information can be downloaded at: https://www.mdpi.com/article/10.3390/cells12010057/s1, Figure S1: Observations vs. individual prediction plots for the concentration-response curves for CIS and CIM as single agents in Huh7 and MDA-MB-468 cancer cell lines; Figure S2: Observations vs. individual prediction plots for the cell viability (PD model) fittings for CIS, CIM, and CIS+CIM in Huh7 and MDA-MB-468.
