# Supplementary material for: Pharmacodynamic Modeling to Evaluate the Impact of Cimetidine, an OCT2 Inhibitor, on the Anticancer Effects of Cisplatin
Source: Cells. 2022 Dec 23;12(1):57. doi: 10.3390/cells12010057 (PMC9818342; doi:10.3390/cells12010057)
Supplement: Supplementary file 1 [file cells-12-00057-s001.zip › cells-2053353-supplementary.pdf]

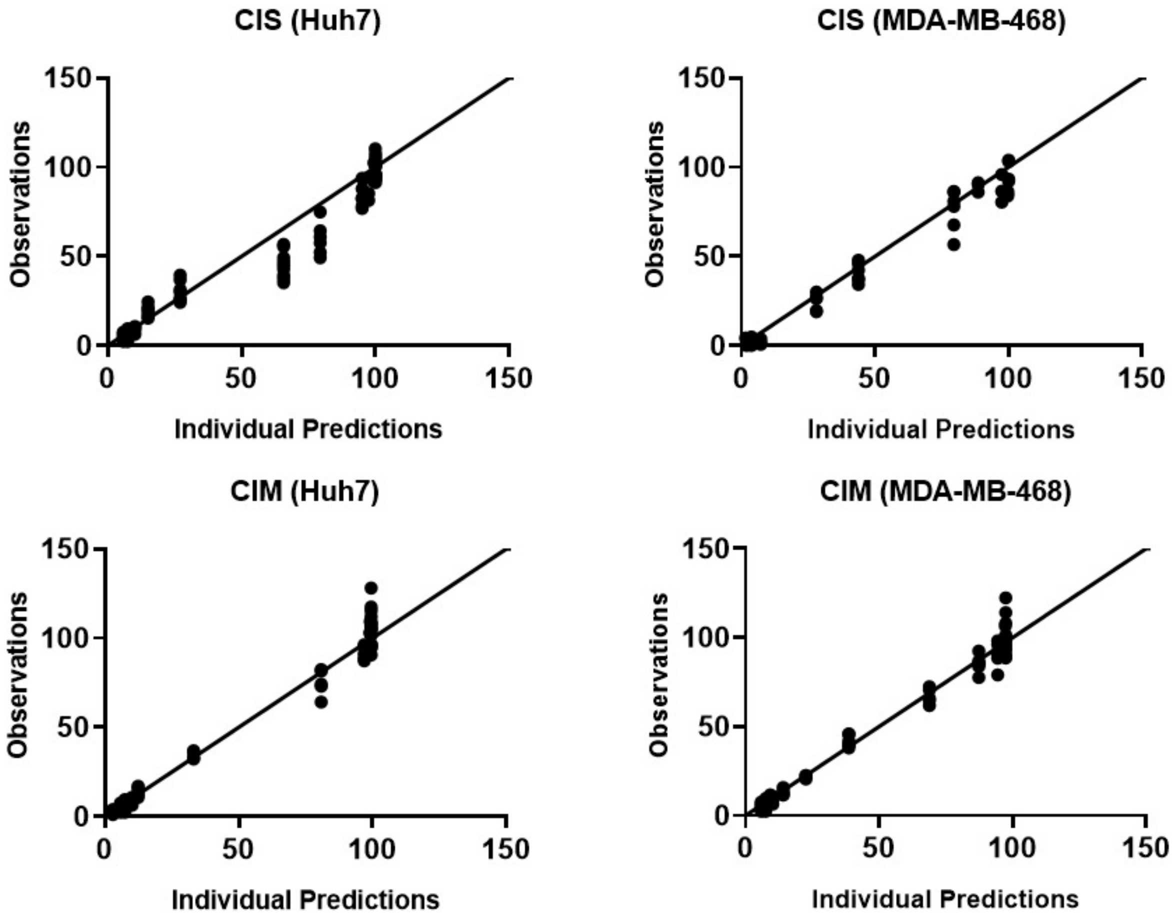

Figure S1. Observations vs. individual prediction plots for the concentration-response curves for CIS (*top*) and CIM (*bottom*) as single agents in Huh7 (*left*) and MDA-MB-468 (*right*) cancer cell lines. The black solid circles represent observed data while the solid line represents the identity line with y=x.

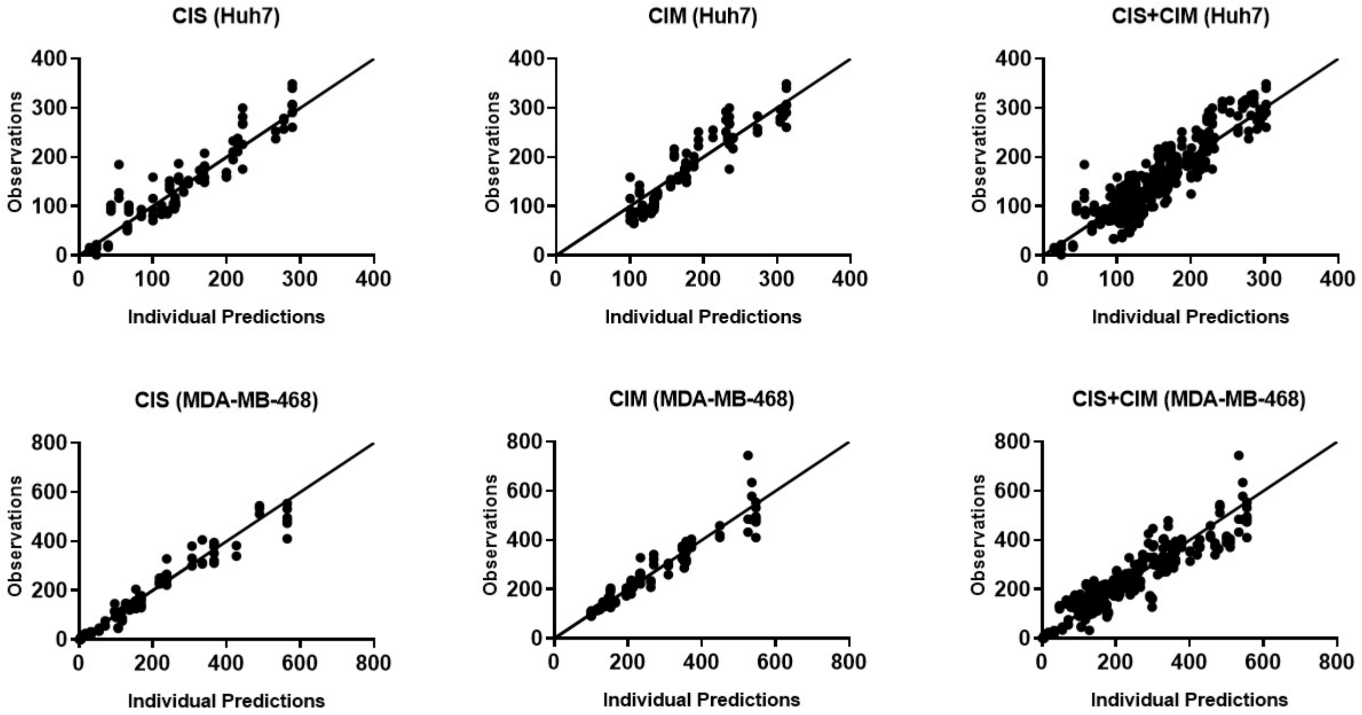

Figure S2. Observations vs. individual prediction plots for the cell viability (PD model) fittings for CIS (*left*), CIM (*middle*), and CIS+CIM (*right*) in Huh7 (*top*) and MDA-MB-468 (*bottom*). The black solid circles represent observed data while the solid line represents the identity line with y=x.
